# Natural defense against colorectal cancer: the effects of kaempferol on epigenetics, apoptosis, inflammation, oxidative stress, and cell proliferation

**DOI:** 10.3389/fphar.2026.1775563

**Published:** 2026-06-25

**Authors:** Jinglin Chen, Qingqing Yu, Yanting Sun

**Affiliations:** 1 Department of Clinical Laboratory, Shiyan People’s Hospital (Affiliated People’s Hospital of Hubei University of Medicine), Shiyan, Hubei, China; 2 Biomedical Research Institute, Hubei University of Medicine, Shiyan, Hubei, China; 3 Independent Researcher, Nanjing, Jiangsu, China

**Keywords:** colorectal cancer, inflammation, invasion, kaempferol, proliferation

## Abstract

Colorectal cancer (CRC) remains a leading cause of cancer-related morbidity and mortality worldwide, driven by complex genetic, epigenetic, and environmental factors. Among natural bioactive compounds, kaempferol (a dietary flavonoid found abundantly in fruits, vegetables, and medicinal plants) has garnered significant attention for its promising anticancer properties. Accumulating evidence suggests that kaempferol exerts multifaceted effects on CRC through modulation of key molecular signaling pathways. These pathways regulate critical cellular processes such as proliferation, invasion, inflammation, angiogenesis, oxidative stress, and apoptosis. Moreover, kaempferol has shown the ability to reverse drug resistance, modulate epigenetic mechanisms, and enhance the efficacy of conventional chemotherapeutics, positioning it as a potential adjuvant therapy in CRC treatment. This review provides an updated overview of the molecular mechanisms underlying the anticancer effects of kaempferol in CRC and highlights the current challenges in translating these findings into clinical applications.

## 1 Introduction

Colorectal cancer (CRC) remains one of the most formidable health challenges globally, both in terms of incidence and mortality. According to the most recent global estimates, CRC ranks as the third most commonly diagnosed malignancy and is the second leading cause of cancer-related deaths. As of 2022, more than 1.9 million new CRC cases were reported, and projections suggest that this number may exceed 2.4 million by 2035 (Bray et al., 2018; Sung et al., 2021). These rising figures are driven not only by aging populations but also by the growing prevalence of risk factors related to lifestyle and diet. Sedentary behavior, high consumption of red and processed meats, alcohol intake, smoking, and low fiber intake have been consistently linked to an elevated CRC risk. Conversely, diets abundant in fruits, vegetables, and phytochemicals appear to exert a protective effect (Baena and Salinas, 2015). Among the most promising of these is kaempferol, a polyphenolic flavonol found in numerous edible plants including apples, onions, kale, broccoli, grapes, and tea. Additionally, it is abundant in several medicinal plants such as Ginkgo biloba, Sophora japonica, and Moringa oleifera. Kaempferol is not only widespread in human diets but also exhibits an impressive range of pharmacological properties. These include antioxidant, anti-inflammatory, antimicrobial, cardioprotective, neuroprotective, and notably, anticancer effects (Calderón-Montaño et al, 2011; Wang et al., 2018). Epidemiological studies suggest an inverse association between kaempferol intake and the risk of several cancers, including CRC (Qattan et al., 2022). These findings are supported by preclinical studies demonstrating its ability to modulate multiple cancer-related signaling pathways (Arai et al., 2000).

One of the key features of kaempferol is its ability to selectively inhibit the growth of malignant cells while sparing or even protecting normal cells. Through both intrinsic and extrinsic pathways, kaempferol promotes programmed cell death by activating key molecular effectors such as p53, caspases, and b-cell lymphoma 2 (BCL-2) family proteins (Rahman et al., 2021). For instance, kaempferol has been shown to increase the expression of p53-upregulated modulator of apoptosis (PUMA) and to trigger the release of cytochrome c from mitochondria (Rajendran et al., 2014). Moreover, it enhances the activation of caspase-3 and PARP cleavage, hallmarks of apoptosis (Al Sabaani, 2020). Inflammation is another hallmark of CRC, with pathways such as nuclear factor kappa B (NF-κB), cyclooxygenase-2 (COX-2), and interleukin-6 playing pivotal roles in tumor promotion and immune evasion. Kaempferol effectively downregulates these pro-inflammatory mediators and interferes with associated signaling pathways. At the level of cell proliferation, kaempferol interferes with key regulatory proteins governing the cell cycle. It inhibits cyclin-dependent kinases (CDKs), particularly CDK2, leading to cell cycle arrest at the G1 or G2/M phase depending on the cellular context.

Kaempferol may also enhance the therapeutic efficacy of conventional chemotherapeutic agents, including 5-fluorouracil (5-FU), by modulating key survival pathways such as phosphoinositide 3-kinase (PI3K)/protein kinase B (AKT), mitogen-activated protein kinase (MAPK), and Janus Kinase (JAK)/signal transducer and activator of transcription 3 (STAT3), as well as regulating cancer-associated microRNAs (miRNAs) involved in metabolic reprogramming (Gmeiner and Okechukwu, 2023; Riahi-Chebbi et al., 2019; Wu et al., 2021). Overall, kaempferol shows strong potential for CRC management due to its multi-targeted biological activities and low toxicity. Its ability to interfere at different stages of tumor development supports its role as both a preventive and therapeutic candidate. As a naturally occurring dietary compound, it may also serve as a supportive agent alongside standard treatments in combination strategies designed to improve therapeutic outcomes (Amjad et al., 2022). Given the urgent need for safer and more effective interventions in CRC, further investigation into molecular mechanisms of kaempferol is warranted. This review aims to systematically summarize the role of kaempferol in regulating apoptosis, epigenetic modifications, oxidative stress, inflammation, and cell proliferation in CRC.

## 2 Methodology of the review

Studies included human CRC cell lines (e.g., HCT116, HT29, SW480, RKO) and *in vivo* animal models. This narrative review was conducted through a comprehensive literature search using electronic databases including PubMed, Scopus, Web of Science, and Google Scholar. Relevant studies published in English up to 2025 were identified using combinations of the following keywords: “kaempferol”, “colorectal cancer”, “oxidative stress”, “apoptosis”, “inflammation”, “epigenetics”, “cell proliferation”, and “signaling pathways”. Original research articles, experimental studies (*in vitro* and *in vivo*), and relevant review articles focusing on the molecular and therapeutic effects of kaempferol in CRC were included. Articles not related to CRC or lacking sufficient mechanistic relevance were excluded. The collected studies were critically evaluated and categorized according to the major biological mechanisms discussed in this review, including oxidative stress regulation, apoptosis, inflammation, epigenetic modulation, and cell proliferation pathways.

No artificial intelligence tools were used in the writing, analysis, interpretation, or preparation of this manuscript.

## 3 Kaempferol: sources, bioavailability, and general anticancer promise

### 3.1 Natural occurrence and dietary sources

Over the past several decades, naturally occurring bioactive compounds derived from plants have attracted significant attention for their therapeutic potential, including anticancer effects. Among these, kaempferol, a flavonol widely distributed in edible plants, has emerged as a compound of particular interest due to its low toxicity and diverse biological activities (Bangar et al., 2023; Rice-Evans, 2001). Kaempferol is a polyphenolic compound found in both free and glycosylated forms, commonly bound to sugars such as glucose, rhamnose, or rutinoside (Cid-Ortega and Monroy-Rivera, 2018; Periferakis et al., 2022). It is widely present in fruits and vegetables, including apples, onions, citrus fruits, spinach, kale, broccoli, tea, and berries (Jeganathan et al., 2016; Zhang et al., 2010). Higher concentrations have been reported in capers, while leafy vegetables such as spinach and cabbage contain moderate levels (Chen et al., 2023). In addition to dietary sources, kaempferol is also present in medicinal plants used in Traditional Chinese Medicine (TCM) and Ayurveda (Mazza and Oomah, 2000; Pietta et al., 2003). TCM have contributed to the identification of kaempferol-rich plants, which are now being systematically investigated for their pharmacological activities (Sokal et al., 2024).

### 3.2 Absorption, metabolism, and bioavailability

The bioavailability of kaempferol has been a subject of intensive research due to its relevance in determining the clinical efficacy of dietary flavonoids. Upon ingestion, kaempferol is primarily absorbed in the small intestine. Its aglycone form is moderately lipophilic, enabling passive diffusion across intestinal membranes. However, studies suggest that both active transport mechanisms and facilitated diffusion may also play a role, depending on the specific glycosidic form consumed (Ma et al., 2017; Viskupičová et al., 2008). Experimental studies indicate that the oral absorption of kaempferol is relatively limited, with estimated intestinal absorption ranging between approximately 2% and 20%, depending on the administered form, food matrix, and experimental model (Dabeek and Marra, 2019; Manach et al., 2005). A substantial proportion of ingested kaempferol therefore remains unabsorbed in the gastrointestinal tract and becomes available for microbial metabolism in the colon. Following absorption, kaempferol undergoes extensive phase II metabolism in the intestinal epithelium and liver, producing glucuronidated, methylated, and sulfated conjugates that can be detected in systemic circulation (Chen and Chen, 2013; Dabeek and Marra, 2019; Manach et al., 2005). These metabolites may retain or even enhance some of the biological activities of the parent compound. Studies have further demonstrated that only low plasma concentrations of free kaempferol are typically achieved after oral intake, whereas conjugated metabolites represent the predominant circulating forms (Calderón-Montaño et al., 2011; A. Y. Chen and Chen, 2013). Interindividual differences in gut microbiota composition, metabolic enzyme activity, dietary patterns, and intestinal transit time may substantially influence its absorption efficiency and systemic exposure (Calderón-Montaño et al., 2011; Chen and Chen, 2013). Although tissue accumulation of kaempferol metabolites has been observed in organs such as the liver and kidney, this accumulation does not necessarily indicate high systemic bioavailability. Rather, it reflects tissue distribution, metabolic conversion, and local retention of kaempferol-derived metabolites following repeated exposure (Barve et al., 2009). In renal cells, kaempferol may even contribute to the biosynthesis of the ubiquinone (Coenzyme Q) ring, implicating a potential mechanism for its antioxidant action (Barve et al., 2009).

### 3.3 Anticancer potential and biological activities

Kaempferol has demonstrated a wide range of anticancer properties across various *in vitro* and *in vivo* models. It exerts its effects through multiple mechanisms that include antioxidant, anti-inflammatory, antiproliferative, pro-apoptotic, anti-angiogenic, and anti-metastatic actions (Ashrafizadeh et al., 2020). These multifaceted effects are largely attributed to its ability to interact with key signaling molecules and transcription factors involved in cancer progression. Studies have shown that kaempferol inhibits cancer cell proliferation by inducing cell cycle arrest—often at the G1 or G2/M phases—and by promoting programmed cell death via both intrinsic and extrinsic apoptotic pathways (Gao et al., 2018). It regulates tumor suppressor and apoptotic proteins such as tumor protein p53 (TP53), cyclin-dependent kinase inhibitor 1A, BCL-2, and survivin. In addition, kaempferol suppresses angiogenesis by downregulating vascular endothelial growth factor expression (Kashafi et al., 2017; Luo et al., 2012). Kaempferol also modulates cancer cell metabolism by regulating glycolysis-related enzymes and cancer-associated miRNAs, thereby disrupting energy production and enhancing chemosensitivity (Shuvalov et al., 2023; Yao et al., 2016). Moreover, kaempferol has been documented to exhibit a synergistic effect when combined with standard chemotherapeutic agents such as 5-FU through the modulation of various molecular pathways that are implicated in cell survival, apoptosis, and metabolic regulation, which include the PI3K/AKT, MAPK, and JAK/STAT3 signaling cascades. These interactions may serve to augment chemosensitivity in CRC cells that exhibit resistance, while concurrently diminishing the necessary therapeutic dosage and the associated toxicity (Chen and Chen, 2013; Imran et al., 2019; To and Cho, 2021). Due to its multi-targeted mechanisms and favorable safety profile, kaempferol represents a promising candidate for cancer prevention and therapeutic development (Shahbaz et al., 2023). In conclusion, kaempferol represents a promising nutraceutical compound in CRC research, with additional evidence reported in other cancer types, including breast, lung, prostate, and gastric cancers. Its multitargeted actions against cancer hallmarks, coupled with its abundance in everyday foods and herbs, make it an appealing subject for further preclinical and clinical investigation.

## 4 Apoptosis and CRC

Apoptosis is a tightly regulated process through which cells are eliminated in an orderly manner, helping to maintain tissue homeostasis. Unlike necrosis, which often results in inflammation and damage to surrounding tissues, apoptosis involves controlled breakdown of cellular components, followed by removal of the resulting fragments by neighboring cells. This natural form of cell death is essential not only during growth and development but also for immune regulation and protection against diseases, including cancer (Santavanond et al., 2021; Shahbaz et al., 2023). In CRC, this balance between cell survival and programmed cell death is disrupted. A key hallmark of CRC is reduced apoptotic activity, which allows abnormal cells to survive longer. Over time, these cells accumulate mutations, leading to uncontrolled proliferation and resistance to therapies such as chemotherapy and radiotherapy (Bedi et al., 1995; Brown and Wilson, 2003). At the cellular level, apoptosis occurs mainly through two pathways: the intrinsic (mitochondrial) pathway and the extrinsic (death receptor–mediated) pathway (Hongmei, 2012). The intrinsic route is governed by the BCL-2 family of proteins, which includes both pro-apoptotic and anti-apoptotic members. When the pro-apoptotic proteins, such as BCL-2-associated X protein (BAX) or BCL-2 antagonist/killer protein, are activated, they cause the mitochondria to release cytochrome c. This molecule then triggers the assembly of a protein complex called the apoptosome, which activates caspase-9, a crucial enzyme that initiates a series of reactions leading to cell death (Boland and Goel, 2010; Brentnall et al., 2013). In colorectal tumors, an imbalance in these regulatory proteins is often observed. Anti-apoptotic proteins like BCL-2 are commonly found in high levels, while their pro-apoptotic counterparts are underexpressed. This shift in favor of survival signals gives tumor cells an advantage, making them more resistant to apoptosis and therefore harder to treat. In fact, BCL-2 overexpression has been identified in a large percentage of CRC cases and is frequently associated with recurrence and poor clinical outcomes (Huang et al., 2017). The extrinsic pathway, meanwhile, relies on surface receptors such as Fas cell surface death receptor (FAS) and tumor necrosis factor-related apoptosis-inducing ligand (TRAIL) receptors of death receptor 4 (DR4) and death receptor 5 (DR5). When these receptors bind to their respective ligands, they trigger the activation of caspase-8, another key enzyme in the apoptosis process. However, similar to the intrinsic pathway, disruptions in this signaling route are also common in CRC and can limit the effectiveness of certain therapies designed to trigger this cell death mechanism (Hector and Prehn, 2009).

In addition to classical apoptotic pathways, other forms of programmed cell death have been described in CRC, including caspase-independent mechanisms such as apoptosis-inducing factor-mediated death and autophagy-associated cell death (Dinicola et al., 2010). These alternative pathways may contribute to therapy resistance and represent potential therapeutic targets. One of the major challenges in CRC treatment is resistance to therapy, which is closely linked to impaired apoptotic signaling. Alterations in key regulators such as caspases, TP53 mutations, and activation of survival pathways including PI3K/AKT and NF-κB enable cancer cells to evade apoptosis and continue proliferating despite treatment (Zhang and Yu, 2013). To overcome this, therapeutic strategies are being developed to restore apoptotic signaling. These include targeting anti-apoptotic proteins, activating death receptors, and re-engaging caspase-dependent pathways. In addition, apoptosis-related molecules are being investigated as predictive biomarkers for treatment response, supporting more personalized therapeutic approaches (Mishra et al., 2013). In summary, dysregulation of apoptosis plays a central role in CRC initiation, progression, and therapy resistance. A deeper understanding of apoptotic signaling in CRC is essential for the development of more effective and targeted anticancer therapies.

### 4.1 Modulation of apoptotic pathways by kaempferol in CRC: a mechanistic perspective

Apoptosis, a form of programmed cell death, is essential for maintaining tissue integrity and eliminating damaged or malignant cells. In CRC, evasion of apoptosis is a hallmark that contributes to tumor growth, treatment resistance, and poor prognosis (Payne et al., 1995). Increasing evidence shows that natural compounds, particularly kaempferol, modulate apoptotic signaling pathways in CRC cells. One of the key apoptosis-inducing mechanisms involves the mitochondrial (intrinsic) pathway. Li et al. (2009) demonstrated that kaempferol activates this pathway in HCT116 colon cancer cells by inducing phosphorylation of ataxia telangiectasia mutated and upregulating TP53, leading to mitochondrial release of cytochrome c. This event promotes activation of caspase-9 and downstream cleavage of caspase-3, triggering apoptosis. Furthermore, kaempferol enhances expression of PUMA, a pro-apoptotic BCL-2 family member, contributing to mitochondrial dysfunction and apoptotic execution. The extrinsic pathway, driven by death receptor signaling, is also targeted by kaempferol. Yoshida et al. (2008) showed that kaempferol significantly enhances TRAIL-induced apoptosis in SW480 colon cancer cells through upregulation of DR4 and DR5. Among these receptors, DR5 plays a critical role, as its silencing markedly reduces kaempferol–TRAIL–induced apoptosis. Importantly, this combination exhibits minimal toxicity toward normal hepatocytes and blood cells, indicating tumor selectivity. Mitochondrial dysfunction represents a central mechanism underlying kaempferol-induced apoptosis. Bianchini et al. (2024) showed that a methanolic extract of *Diospyros kaki*, rich in kaempferol derivatives, disrupts mitochondrial respiration in CRC cells, leading to increased ROS production and apoptosis. Notably, this effect was selective for tumor cells, with minimal impact on normal colonic epithelium.

The role of oxidative stress in triggering apoptosis was further elucidated by Choi et al. (2018), who revealed that kaempferol-induced apoptosis in HCT116 and SW480 cells involves reactive oxygen species (ROS) generation, followed by activation of p38 MAPK and TP53. ROS scavengers and inhibitors of p38 or caspases were able to block these effects, confirming the central role of the ROS–p38–caspase axis in kaempferol-mediated apoptosis. Lee et al. (2014) further confirmed that kaempferol increases mitochondrial membrane permeability and promotes cytosolic release of cytochrome c in HT-29 cells. This is accompanied by activation of caspases-9, -3, and -7. In addition, kaempferol downregulates anti-apoptotic B-cell lymphoma-extra-large (BCL-XL) while upregulating pro-apoptotic BCL2-interacting killer and BCL2-associated agonist of cell death, thereby shifting the balance toward apoptosis. Activation of the extrinsic pathway was also observed through increased Fas ligand expression and caspase-8 activation. *In vivo* evidence further supports these findings. Hassan, Hassanein and Ahmed (2021) demonstrated that kaempferol combined with fluoxetine enhances caspase-3 activity and reduces oxidative stress and inflammatory markers, including β-catenin and COX-2, in a rat model of colon carcinogenesis. Histological analysis confirmed improved tissue architecture and increased apoptotic activity. Beyond individual pathways, Budisan et al. (2019) showed that kaempferol exerts broad regulatory effects on cell survival networks. In RKO and HCT116 cells, kaempferol inhibited proliferation and invasion while inducing both apoptosis and autophagy. Gene expression analysis revealed modulation of both coding and noncoding RNAs involved in tumor progression. Collectively, these studies demonstrate that kaempferol induces apoptosis in CRC through multiple interconnected mechanisms. These include mitochondrial dysfunction, ROS generation, activation of p53 and MAPK signaling, and engagement of death receptor pathways. Importantly, these effects are largely selective for cancer cells, with minimal toxicity to normal tissues, supporting its therapeutic potential (Table 1). Therefore, kaempferol may represent a promising candidate for combination therapy or dietary-based intervention strategies in CRC.

**TABLE 1:** Modulation of apoptotic pathways by kaempferol in CRC.

Disease/Model	Study type	Treatment	Mechanism of apoptosis	Main findings	Ref
CRC (E705, SW480 cells)	Preclinical (*in vitro*)	D. kaki methanolic extract (kaempferol and quercetin derivatives)	ROS accumulation, mitochondrial dysfunction, apoptosis induction	Selective apoptosis in CRC cells via mitochondrial disruption	Bianchini et al. (2024)
CRC (HCT116 cells)	Preclinical (*in vitro*)	Kaempferol	ATM/p53 pathway activation, cytochrome c release, caspase-3 cleavage, PUMA upregulation	p53-dependent apoptosis and growth inhibition	Li et al. (2009)
CRC (SW480 cells)	Preclinical (*in vitro*)	Kaempferol + TRAIL	DR5/DR4 receptor upregulation, enhanced TRAIL sensitivity	Synergistic apoptosis with kaempferol and TRAIL; no effect on normal cells	Yoshida et al. (2008)
DMH-induced colon cancer in rats	Preclinical (*in vivo*)	Fluoxetine + Kaempferol/EGCG	↑ Caspase-3, ↓ β-catenin, MDA, COX-2, PCNA	Enhanced apoptosis and reduced preneoplastic lesions	Hassan et al. (2021)
CRC (RKO, HCT-116 cells)	Preclinical (*in vitro*)	Kaempferol and CAPE	Gene expression changes, caspase activation, autophagy	Kaempferol induced apoptosis and altered gene mutations	Budisan et al. (2019)
CRC (HCT116, HCT15, SW480 cells)	Preclinical (*in vitro*)	Kaempferol	ROS generation, p38 MAPK and p53 signaling, caspase activation	ROS-dependent apoptosis via p38/p53/caspase cascade	Choi et al. (2018)
CRC (HT-29 cells)	Preclinical (*in vitro*)	Kaempferol	Mitochondrial cytochrome c release, FAS signaling, caspases, Bcl-xL downregulation	Induced mitochondrial and death receptor-mediated apoptosis	Lee et al. (2014)

## 5 Epigenetics and CRC

CRC develops not only through genetic mutations but also through significant epigenetic alterations that regulate gene expression without changing the DNA sequence. These modifications include DNA methylation, histone modifications, and non-coding RNAs, which collectively control gene expression and cellular function (Deans and Maggert, 2015). A key advantage of targeting epigenetic mechanisms is their reversibility, unlike genetic mutations, making them attractive for diagnostic and therapeutic applications. Among the most extensively studied epigenetic mechanisms in CRC is DNA methylation. In normal cells, CpG islands—regions enriched in cytosine and guanine—located near gene promoters are typically unmethylated, allowing active gene transcription (Esteller and Herman, 2002). In CRC, however, promoter hypermethylation frequently leads to silencing of tumor suppressor genes and DNA repair genes. At the same time, global DNA hypomethylation is commonly observed in non-promoter regions (Bardhan and Liu, 2013). This dual alteration contributes to genomic instability and activation of normally silenced repetitive elements such as LINE-1 sequences, thereby promoting tumor initiation and progression (Estécio et al., 2007). Reduced LINE-1 methylation has also been investigated as a potential biomarker for CRC risk and tumor aggressiveness (Inamura et al., 2014). The pattern of DNA methylation in CRC changes as the disease advances. Early stages show widespread demethylation, while more advanced stages present with localized hypermethylation at key genes such as MLH1, CDKN2A (p16), adenomatous polyposis coli (APC), MGMT, and TIMP3, which are involved in DNA repair, cell cycle regulation, and tumor suppression (Lao and Grady, 2011; Toyota et al., 1999). This forms a methylation pattern known as the CpG island methylator phenotype (CIMP), where multiple genes are silenced simultaneously (Baba et al., 2010; Bariol et al., 2003). CIMP-positive tumors often show unique molecular features and tend to co-occur with specific genetic mutations, indicating a strong interplay between genetic and epigenetic mechanisms in CRC (Tapial et al., 2019). Histone modification represents another important epigenetic regulatory mechanism in CRC. Histones are structural proteins around which DNA is wrapped to form chromatin, and their post-translational modifications, such as acetylation and methylation, regulate chromatin accessibility and gene transcription. In general, histone acetylation is associated with transcriptional activation, whereas certain histone methylation marks are linked to gene repression. In CRC, aberrant histone modifications can lead to silencing of tumor suppressor genes or activation of oncogenic pathways (An et al., 2023). Non-coding RNAs, particularly miRNAs, also play a critical role in CRC progression by regulating gene expression at the post-transcriptional level. These short RNA molecules bind to messenger RNAs (mRNAs), resulting in translational repression or mRNA degradation. Dysregulation of miRNA expression disrupts normal cellular homeostasis and contributes to uncontrolled proliferation and resistance to apoptosis (Yang et al., 2022). Collectively, these epigenetic alterations act in a coordinated manner to regulate CRC initiation and progression. Because these modifications are potentially reversible, epigenetic mechanisms represent promising targets for diagnostic, prognostic, and therapeutic strategies in CRC.

### 5.1 Epigenetic targets of kaempferol in CRC: from miRNA suppression to metastatic inhibition

**FIGURE 1:**
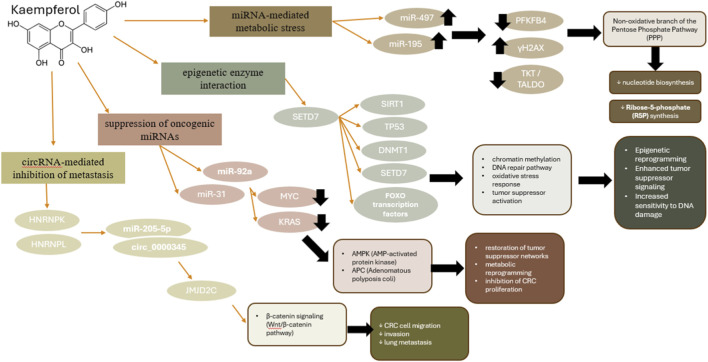
Epigenetic and metabolic mechanisms by which kaempferol suppresses colorectal cancer progression. Kaempferol regulates multiple epigenetic, metabolic, and non-coding RNA pathways to inhibit CRC growth and metastasis. (1) miRNA-mediated metabolic stress: Kaempferol upregulates miR-195 and miR-497, which suppress PFKFB4, leading to inhibition of the non-oxidative pentose phosphate pathway and downstream enzymes TKT and TALDO. This results in reduced ribose-5-phosphate (R5P) synthesis, impaired nucleotide biosynthesis, elevated γH2AX levels, and DNA damage–induced cell-cycle arrest. (2) Epigenetic enzyme interaction: Kaempferol binds SETD7, disrupting signaling networks involving DNMT1, TP53, SIRT1, and FOXO transcription factors, promoting chromatin reprogramming, enhanced tumor-suppressor activity, and increased sensitivity to DNA damage. (3) Suppression of oncogenic miRNAs: Kaempferol downregulates oncogenic miR-31 and miR-92a, reducing KRAS and c-MYC expression while restoring tumor suppressors AMPK and APC, contributing to inhibition of CRC proliferation and metabolic reprogramming. (4) circRNA-mediated inhibition of metastasis: Kaempferol interacts with RNA-binding proteins HNRNPK and HNRNPL, suppressing circ_0000345, which releases miR-205-5p from circRNA sponging. miR-205-5p downregulates JMJD2C, leading to inhibition of β-catenin/Wnt signaling and reduced CRC cell migration, invasion, and lung metastasis. Collectively, kaempferol orchestrates a coordinated multi-target epigenetic, metabolic, and non-coding RNA network resulting in suppression of tumor progression and metastasis.

The link between epigenetic dysregulation and CRC has become increasingly evident, particularly through mechanisms involving miRNAs, DNA repair suppression, and non-coding RNAs such as circular RNAs. Several studies indicate that kaempferol modulates epigenetic and metabolic pathways contributing to DNA damage and tumor suppression in CRC cells. Wu et al. (2025) showed that kaempferol induces DNA damage in CRC cells by targeting the nonoxidative branch of the pentose phosphate pathway (PPP). Kaempferol treatment increases γH2AX levels, a marker of DNA damage, and induces DNA strand breaks confirmed by comet assays. This effect is associated with suppression of ribose-5-phosphate (R5P) synthesis, a key substrate for nucleotide biosynthesis, thereby impairing DNA integrity in rapidly proliferating tumor cells. Mechanistically, this inhibition occurs through upregulation of miR-195 and miR-497, which target PFKFB4, a key regulator of the nonoxidative PPP. Consequently, downstream enzymes such as transketolase (TKT) and transaldolase (TALDO) are suppressed, leading to enhanced DNA damage and cell cycle arrest. Priyamvada et al. (2025) used computational modeling to demonstrate that kaempferol binds strongly to SET domain-containing lysine methyltransferase 7 (SETD7), a histone methyltransferase involved in chromatin regulation. This interaction may disrupt SETD7-associated signaling networks involving DNA (cytosine-5)-methyltransferase 1 (DNMT1), TP53, Sirtuin 1, and Forkhead box O transcription factors (FOXO) proteins, which are critical regulators of epigenetic control, DNA repair, and oxidative stress responses. These findings suggest that kaempferol exerts multi-target regulatory effects across epigenetic and stress-response pathways in CRC. Beyond DNA damage, kaempferol also regulates miRNA expression, influencing both oncogenes and tumor suppressor genes. Gutierrez-Uribe et al. (2020) reported that kaempferol-3-O-glycoside from black beans significantly downregulates miR-31 and miR-92a, both of which are overexpressed in CRC and associated with Kirsten rat sarcoma viral oncogene homolog (KRAS) and MYC oncogenic signaling. Following kaempferol treatment, expression of KRAS and c-MYC was reduced, while tumor suppressor genes such as AMPK and APC were upregulated. This indicates that kaempferol suppresses oncogenic miRNAs while restoring tumor-suppressive gene networks. In terms of metastatic regulation, kaempferol interferes with circRNA-mediated signaling pathways. Pu et al. (2024) showed that kaempferol directly interacts with RNA-binding proteins HNRNPK and HNRNPL, leading to downregulation of circ_0000345, a circRNA associated with CRC metastasis. Circ_0000345 acts as a sponge for miR-205-5p, thereby enhancing JMJD2C expression and activating β-catenin signaling. By reducing circ_0000345 levels, kaempferol restores miR-205-5p activity, suppresses JMJD2C, and inhibits β-catenin signaling, ultimately reducing CRC cell migration and lung metastasis *in vitro* and *in vivo*. Collectively, these findings demonstrate that kaempferol exerts broad epigenetic and metabolic regulatory effects in CRC, supporting its potential as a candidate for chemopreventive and therapeutic applications (Table 2; Figure 1).

**TABLE 2:** Epigenetic targets of kaempferol in colorectal cancer.

Study type	Kaempferol treatment	Mechanisms	Key results	Ref
Pre-clinical (*in vitro* and *in vivo*)	Kaempferol (modulates miR-195/497 and PPP)	Inhibition of nonoxidative PPP; miR-195/497 upregulation; PFKFB4 downregulation; DNA damage	DNA damage induction confirmed via γH2AX and comet assay; restoration by nucleoside supplementation	Wu et al. (2025)
*In silico* and computational modeling	Kaempferol (targeted docking and network modeling)	Multi-target binding: SETD7-DNMT1 and FOXO signaling; supports FOXO-mediated epigenetic control	SETD7 showed highest binding affinity; PPI with epigenetic regulators; proposes epigenetic synergy	Priyamvada et al. (2025)
Pre-clinical (*in vitro*, RKO cells)	Kaempferol-3-O-glycoside (1 mM, 72h)	Downregulation of miR-31 and miR-92a; reduced KRAS, c-MYC; increased AMPK, APC	Significant decrease in oncogenic miRNAs; tumor suppressors upregulated	Gutierrez-Uribe et al. (2020)
Pre-clinical (*in vitro* and *in vivo*)	Kaempferol (targets circ_0000345/miR-205-5p)	Suppression of circ_0000345; miR-205-5p rescue; JMJD2C/β-catenin pathway inhibition	Inhibited CRC metastasis; disrupted circRNA-miRNA axis and epigenetic co-regulators HNRNPK/HNRNPL	Pu et al. (2024)

## 6 Kaempferol-induced cell cycle arrest in CRC: molecular insights and therapeutic potential

**FIGURE 2:**
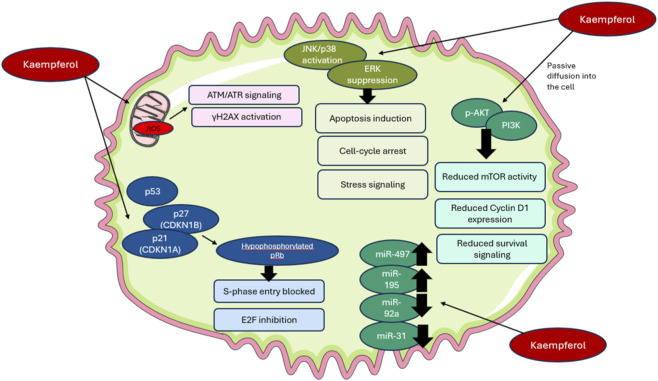
Kaempferol-induced cell-cycle arrest in colorectal cancer cells. Kaempferol enters colorectal cancer (CRC) cells through passive diffusion and triggers multiple molecular events that converge on cell-cycle arrest, stress signaling, and apoptosis. Inside the mitochondria, kaempferol elevates reactive oxygen species (ROS), activating ATM/ATR and γH2AX, which promote p53 stabilization. Activated p53 induces the CDK inhibitors p21 (CDKN1A) and p27 (CDKN1B), leading to hypophosphorylation of pRb, suppression of E2F activity, and blockade of S-phase entry. Kaempferol simultaneously modulates MAPK pathways by activating JNK/p38 and suppressing ERK, resulting in enhanced apoptosis, cell-cycle arrest, and stress-response signaling. In parallel, kaempferol inhibits the PI3K/p-AKT axis, reducing mTOR activity, decreasing Cyclin D1 expression, and weakening survival signaling. Kaempferol also regulates key CRC-related microRNAs by upregulating miR-195 and miR-497, while downregulating oncogenic miR-92a and miR-31, further reinforcing growth suppression. Collectively, these coordinated actions converge to inhibit proliferation and promote programmed cell death in CRC cells.

CRC is characterized by uncontrolled cellular proliferation resulting from dysregulated cell cycle progression (Testa et al., 2018). Natural flavonoids, such as kaempferol, have emerged as potential agents capable of modulating cell cycle checkpoints and suppressing tumor growth. Accumulating evidence indicates that kaempferol interferes with cell cycle progression at both the G0/G1 and G2/M checkpoints, leading to reduced CRC cell proliferation. Martineti et al. (2010) investigated kaempferide triglycoside, a glycosylated kaempferol derivative isolated from *Dianthus caryophyllus*, in HCT8 colon cancer cells. The compound inhibited proliferation by inducing G0/G1 phase arrest, independent of classical estrogen receptor activation. In estrogen receptor β-overexpressing cells, treatment further increased antioxidant enzyme activity, suggesting a link between redox regulation and cell cycle control. Xing et al. (2024) examined kaempferol derivatives as CDK2 inhibitors using pharmacophore modeling and molecular dynamics simulations. Several derivatives demonstrated strong and stable binding to CDK2, a key regulator of the G1/S transition. These interactions suggest that kaempferol-based compounds may suppress proliferation by directly targeting cell cycle kinases (Figure 2). Park et al. (2021) studied kaempferol in oxaliplatin-resistant CRC cells and found that it enhanced G2/M phase arrest in resistant HCT116 and HT29 cells. This effect was associated with modulation of AP-1 transcriptional activity, suggesting a role in overcoming chemotherapy resistance. Wu et al. (2021) demonstrated that kaempferol delays G1 phase progression through regulation of miR-339-5p, which influences alternative splicing of pyruvate kinase isoforms via hnRNPA1 and PTBP1. This metabolic shift from PKM2 to PKM1 suppresses aerobic glycolysis and contributes to cell cycle inhibition. Lee et al. (2014) further showed that kaempferol-induced cell cycle arrest is closely linked to apoptotic activation in HT-29 cells. Treatment resulted in mitochondrial dysfunction, chromatin condensation, and activation of caspase-3, -7, and -9, along with downregulation of anti-apoptotic BCL-XL. In addition, increased Fas ligand expression and membrane permeability suggested coordinated activation of apoptotic and cell cycle disruption pathways. Collectively, these findings demonstrate that kaempferol exerts multi-level regulatory effects on CRC cell proliferation. By inhibiting CDK2 activity, modulating miRNA-mediated metabolic pathways, overcoming drug resistance mechanisms, and promoting mitochondrial-dependent apoptosis, kaempferol acts as a multi-target anticancer agent. These findings support further *in vivo* validation and suggest potential application of kaempferol or its derivatives as adjunctive therapies targeting cell cycle regulation in CRC (Table 3).

**TABLE 3:** Kaempferol-induced cell cycle arrest in colorectal cancer.

Disease	Study type	Treatment condition	Mechanism	Key findings	Ref
Colorectal Cancer (HCT8 cells)	Pre-clinical (*in vitro*)	Kaempferide triglycoside from *Dianthus caryophyllus*	G0/G1 arrest, ERβ-related, increased antioxidant enzymes	Cell cycle arrest in G0/G1, enhanced by ERβ overexpression	Martineti et al. (2010)
Colorectal Cancer (*in silico* CDK2 target)	Computational (pharmacophore/docking)	Kaempferol derivatives targeting CDK2	CDK2 inhibition, stable ligand-protein complexes	High binding affinity to CDK2, stable drug-target interactions	Xing et al. (2024)
Oxaliplatin-resistant Colorectal Cancer (HCT116, HT29)	Pre-clinical (*in vitro*)	Kaempferol treatment in OxR cells	G2/M phase arrest, MAPK/PI3K-AKT pathway, AP-1 inhibition	Greater sensitivity in resistant cells, strong proliferation block	Park et al. (2021)
Colorectal Cancer (HCT116, DLD1)	Pre-clinical (*in vitro*)	Kaempferol dose-dependent treatment	miR-339-5p-PKM splicing axis, glycolysis inhibition, G1 delay	Reduced glucose metabolism, increased PKM1/PKM2 switch	Wu et al. (2021)
Colorectal Cancer (HT-29)	Pre-clinical (*in vitro*)	Kaempferol (0–60 μmol/L)	Mitochondrial pathway, caspase cascade, apoptosis induction	Apoptosis via FAS ligand, mitochondrial permeability, Bad upregulation	Lee et al. (2014)

## 7 Other related molecular signaling pathway

### 7.1 Oxidative stress

Oxidative stress plays a significant role in the development and progression of CRC. It results from an imbalance between the generation of ROS such as superoxide, hydroxyl radicals, and hydrogen peroxide—and the body’s antioxidant defense system. While ROS are essential for normal cellular signaling, excessive amounts can cause extensive damage to DNA, lipids, and proteins (Haklar et al., 2001; Janssen et al., 1999). This damage contributes to genomic instability, a hallmark of cancer, and may lead to mutations in critical genes such as p53, APC, BRAF, and KRAS (Lee et al., 2015). Evidence shows that CRC tissues exhibit elevated markers of oxidative stress, including increased levels of 8-hydroxydeoxyguanosine, protein carbonyls, and advanced oxidation protein products. Simultaneously, antioxidant enzyme activities and levels of vitamins C and E are often decreased in CRC patients, indicating impaired redox regulation. These disruptions can influence gene expression and promote cancerous transformation by supporting inflammation and tumor growth (Chang et al., 2008). Although ROS contribute to cancer progression, the reliance of cancer cells on redox imbalance can be used against them. By targeting oxidative stress pathways, treatments may induce cancer cell death, making oxidative stress both a driver of CRC and a therapeutic target.

### 7.2 Kaempferol as a redox-modulating agent in CRC: insights into nuclear factor erythroid 2–related factor 2 (Nrf2) activation and oxidative stress regulation

**FIGURE 3:**
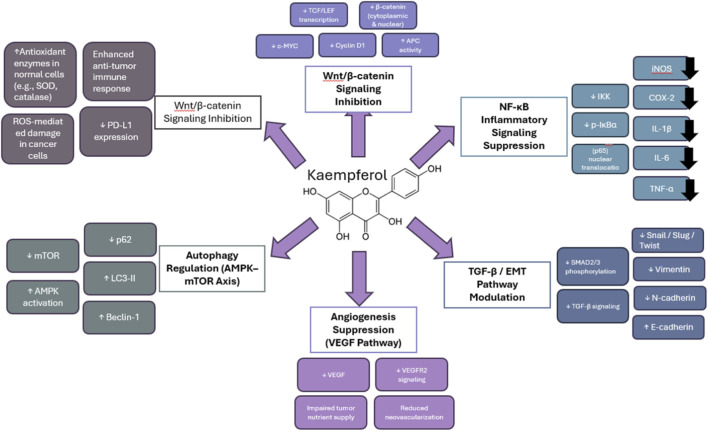
Additional molecular signaling pathways modulated by kaempferol in colorectal cancer. Kaempferol exerts broad anti-tumor activity in colorectal cancer (CRC) by targeting multiple oncogenic and microenvironment-related signaling cascades beyond its epigenetic and cell-cycle effects. Kaempferol inhibits the Wnt/β-catenin pathway, reducing β-catenin stability and nuclear translocation, thereby suppressing TCF/LEF-driven transcription and downstream effectors such as c-MYC and Cyclin D1. Concurrently, kaempferol attenuates NF-κB inflammatory signaling by blocking IKK activation, preventing IκBα degradation, and reducing nuclear NF-κB (p65), leading to decreased expression of pro-inflammatory mediators including TNF-α, IL-6, COX-2, and iNOS. Kaempferol also disrupts TGF-β/SMAD-mediated EMT, promoting epithelial markers (E-cadherin) while repressing mesenchymal drivers (N-cadherin, Vimentin, Snail, Slug, Twist), resulting in reduced invasion and metastatic potential. In addition, kaempferol suppresses angiogenesis by downregulating HIF-1α and VEGF/VEGFR2 signaling, thereby limiting neovascularization. Through activation of AMPK and inhibition of mTOR, kaempferol enhances autophagy-related proteins (Beclin-1, LC3-II) while reducing p62 accumulation, contributing to metabolic stress and tumor suppression. Kaempferol further modulates the tumor microenvironment by reducing oxidative stress imbalance and diminishing immune evasion signals such as PD-L1. Collectively, these interconnected regulatory pathways reinforce kaempferol’s multi-targeted suppression of CRC growth, progression, and therapeutic resistance.

Among the body’s protective mechanisms, the Nrf2 pathway plays a central role in regulating antioxidant responses and reducing oxidative stress. Niestroy et al. (2011) investigated the effects of kaempferol and quercetin on benzo [a]pyrene (BaP)-induced alterations in human colon carcinoma cells, focusing on the aryl hydrocarbon receptor (AhR) and Nrf2 signaling pathways. BaP activated both AhR and Nrf2, leading to increased expression of detoxification enzymes such as CYP1A1 and CYP1B1. Kaempferol, however, attenuated these effects by restoring AhRR expression and suppressing BaP-induced gene activation. In addition, kaempferol modulated Nrf2 signaling and regulated downstream antioxidant genes, including GSTP1 and NQO1, indicating a protective role against ROS-mediated damage. Nirmala and Ramanathan (2011) conducted an *in vivo* study using a 1,2-dimethylhydrazine (DMH)-induced CRC model in rats, comparing kaempferol with irinotecan. Kaempferol significantly reduced lipid peroxidation in liver and blood tissues and restored antioxidant enzyme activities, including catalase, superoxide dismutase, and glutathione peroxidase. These effects were dose-dependent, with the strongest response observed at 200 mg/kg, comparable to irinotecan treatment. Hassanet al. (2021) assessed kaempferol combined with fluoxetine or epigallocatechin gallate in a DMH-induced CRC rat model. Their results demonstrated that kaempferol enhanced antioxidant capacity and exerted anti-inflammatory and pro-apoptotic actions. Although briefly described, these findings further support the complementary role of kaempferol in mitigating oxidative stress and inflammatory damage during the early stages of colorectal carcinogenesis. In another study, Sharma et al. (2024) explored the effects of kaempferol interaction with 5-FU in a CRC model. The compound mitigated 5-FU-induced toxicity by preserving hematological balance and protecting organ integrity. The effects of kaempferol immunomodulatory effects also contributed to reduced oxidative and inflammatory tissue injury, making it a viable candidate for adjunctive therapy to balance efficacy and safety. Altogether, these studies highlight the effects of kaempferol multifaceted antioxidant actions—especially through Nrf2 activation, suppression of lipid peroxidation, and enhancement of endogenous enzyme defense. These properties suggest kaempferol could play a valuable role in future CRC therapeutic strategies focused on redox modulation (Figure 3).

### 7.3 Metabolic rewiring in CRC: the effects of kaempferol in glycolysis and PPP modulation

Metabolic reprogramming is a hallmark of cancer cells, enabling sustained proliferation and resistance to therapy. One of the most prominent alterations is the shift toward aerobic glycolysis, known as the Warburg effect, where cancer cells preferentially convert glucose into lactate even in the presence of oxygen. This metabolic phenotype supports anabolic growth and survival under stress (Chen et al., 2007). Recent studies highlight kaempferol as a regulator of metabolic pathways in CRC, particularly through modulation of glycolysis and the PPP. Wu et al. (2025) demonstrated that kaempferol disrupts the nonoxidative branch of the PPP by targeting PFKFB4 via upregulation of miR-195 and miR-497. This leads to suppression of downstream enzymes such as TKT and TALDO, reducing ribose-5-phosphate availability required for nucleotide biosynthesis. Wu et al. (2021) further showed that kaempferol regulates pyruvate kinase M (PKM) isoforms through miR-339-5p. This miRNA downregulates hnRNPA1 and PTBP1, shifting splicing toward PKM1 rather than PKM2. Since PKM2 supports the Warburg effect, this shift reduces glycolytic flux and promotes oxidative phosphorylation, resulting in decreased glucose uptake, lactate production, and ATP generation. Expanding on glycolytic regulation, Wu et al. (2022) reported that kaempferol reverses 5-FU resistance by targeting PKM2 via miR-326-mediated pathways. This mechanism suppresses PKM2 expression and restores chemotherapy sensitivity by disrupting glycolytic metabolism. Li et al. (2019) provided further evidence of synergism between kaempferol and 5-FU. The combined treatment significantly reduced CRC cell viability, accompanied by increased BAX and decreased BCL-2 and thymidylate synthase expression. In addition, inhibition of the PI3K/AKT pathway suggested involvement of survival signaling in this synergistic anticancer effect. Collectively, these findings demonstrate that kaempferol disrupts metabolic reprogramming in CRC by targeting glycolysis and the PPP through miRNA- and splicing factor–mediated regulation.

### 7.4 Kaempferol as an adjuvant to overcome chemoresistance in CRC

Drug resistance remains a major challenge in CRC therapy, particularly with commonly used agents such as 5-FU and oxaliplatin. Kaempferol, a natural flavonoid, has shown potential in reversing resistance and enhancing therapeutic response. Park et al. (2021) reported that kaempferol significantly inhibited proliferation in oxaliplatin-resistant colon cancer cells by modulating RSK and MAPK signaling pathways. This effect was associated with G2/M phase arrest and suppression of AP-1 activity, suggesting a role in overcoming chemoresistance. In 5-FU-resistant CRC cells, Riahi-Chebbi et al. (2019) demonstrated that kaempferol combined with 5-FU enhanced apoptosis and reduced proliferation. This effect involved inhibition of ROS production and downregulation of STAT3 and AKT phosphorylation, along with restoration of FOXO3A activity, thereby sensitizing resistant cells to chemotherapy. Li et al. (2019) further confirmed the synergistic effect of kaempferol with 5-FU, showing increased apoptosis and reduced expression of thymidylate synthase and p-AKT, both key mediators of chemoresistance. Sharma et al. (2024) additionally reported that kaempferol may mitigate chemotherapy-induced toxicity *in vivo*, where it reduced 5-FU-associated organ damage and helped preserve immune function. Wu et al. (2022) further demonstrated that kaempferol reverses chemoresistance through metabolic regulation by upregulating miR-326, which suppresses PKM2-driven glycolysis, thereby restoring sensitivity to 5-FU. Collectively, these findings indicate that kaempferol acts as a multi-target modulator that enhances chemosensitivity while potentially reducing treatment-related toxicity in CRC.

### 7.5 Anti-metastatic effects of kaempferol in CRC

Kaempferol has shown notable potential in limiting metastasis in CRC through modulation of signaling pathways linked to tumor progression. Pu et al. (2024) found that kaempferol suppresses the JMJD2C/β-CATENIN pathway by interfering with circ_0000345, a circular RNA highly expressed in metastatic CRC tissues. This regulation occurs via miR-205-5p, which is blocked by circ_0000345. Kaempferol restores miR-205-5p function by reducing circ_0000345 levels, thereby lowering β-CATENIN activity and restricting cancer cell migration. In a separate computational study, Sharma et al. (2022) demonstrated that kaempferol can bind effectively to EGFR, HER2, and MEK1—key players in resistance and metastasis of CRC. By targeting these proteins simultaneously, kaempferol may help prevent resistance development and inhibit tumor spread. Together, these studies suggest that kaempferol not only interferes with critical pro-metastatic pathways but may also serve as a valuable multi-targeted compound in advanced CRC therapy.

### 7.6 Immune and inflammatory modulation in CRC: the role of kaempferol

Recent findings suggest that kaempferol plays a regulatory role in the immune and inflammatory environment of CRC, acting on key pathways like NF-κB and COX-2 while also influencing immune checkpoints. Gu et al. (2023) applied network pharmacology and bioinformatics to explore the combined effects of kaempferol and quercetin. Their integrated model revealed that both compounds interact with genes involved in immune regulation and tumor prognosis. The analysis indicated a connection between these gene targets and immune checkpoint markers, suggesting that kaempferol may support immunotherapy in CRC by modulating immune infiltration and checkpoint expression. In earlier animal studies, Hassan et al (2021) reported that co-treatment with kaempferol and fluoxetine attenuated inflammatory markers such as COX-2 and nitric oxide in a rat model of colon carcinogenesis, improving tissue architecture and reducing lesion development.

Additionally, Riahi-Chebbi et al. (2019) demonstrated that kaempferol alone or with 5-FU suppressed NF-κB activity and other survival pathways in resistant CRC cells, reinforcing its potential in overcoming inflammatory-driven resistance. Collectively, these findings indicate that kaempferol exerts anti-metastatic effects in CRC through coordinated regulation of non-coding RNA networks and inhibition of major oncogenic signaling pathways.

### 7.7 Kaempferol enhances gap junction function and differentiation by modulating connexin43 and stat3 pathways

Nakamura et al. (2005) demonstrated that kaempferol can enhance differentiation in partially differentiated colon cancer cells by restoring gap junction intercellular communication (GJIC), a function often impaired in tumor cells. This effect was associated with increased expression and phosphorylation of Connexin43, a key protein that facilitates cell-cell communication. Additionally, kaempferol treatment led to a marked reduction in the phosphorylation of Stat3, a transcription factor involved in proliferation and resistance to apoptosis. The use of a Stat3 inhibitor mimicked the differentiation-inducing effects of kaempferol, suggesting that inhibition of Stat3 plays a critical role in re-establishing GJIC through Connexin43. Collectively, these findings indicate that kaempferol promotes cellular differentiation in CRC cells through Stat3 inhibition and restoration of Connexin43-dependent intercellular communication.

### 7.8 Interconnection of nutrigenomics with kaempferol in CRC

Nutrigenomics investigates the intricate interactions between dietary bioactive compounds and the genomic framework, with a particular emphasis on the manner in which nutrients modulate gene expression, epigenetic mechanisms, and susceptibility to diseases (Ferguson, 2009). Within the domain of CRC, nutrigenomics has garnered heightened scholarly interest due to the capacity of dietary constituents to influence signaling pathways that are critical in tumorigenesis and progression (Romagnolo and Selmin, 2012). Kaempferol, a naturally occurring flavonoid found abundantly in a variety of fruits and vegetables, is posited as a potent nutrigenomic agent owing to its capability to regulate numerous molecular and epigenetic targets pertinent to CRC pathogenesis (Chen and Chen, 2013). Emerging research indicates that kaempferol may affect the trajectory of CRC progression through the modulation of nutrigenomic pathways, which encompass the regulation of miRNAs, DNA methylation patterns, histone modifications, and transcription factors that are integral to processes such as inflammation, oxidative stress, apoptosis, and cellular proliferation (Milenkovic et al., 2013). Kaempferol has been demonstrated to influence critical signaling pathways, including PI3K/AKTt/mTOR, WNT/β-CATENIN, NF-κB, and MAPK, which are intricately associated with gene-diet interactions in CRC (Chen and Chen, 2013; Pan et al., 2010). Moreover, nutrigenomic research suggests that dietary flavonoids such as kaempferol may play a significant role in personalized prevention strategies by altering gene expression profiles and enhancing the efficacy of chemotherapy (Romagnolo and Selmin, 2012). Consequently, elucidating the nutrigenomic implications of kaempferol may yield novel perspectives on personalized nutrition and precision medicine methodologies for the prevention and treatment of CRC.

## 8 Clinical and translational perspectives on kaempferol: pharmacokinetics, safety, and future directions

Kaempferol, a naturally occurring flavonoid found in many plant-based foods, has gained attention for its therapeutic potential in cancer treatment, especially CRC. While laboratory findings show that it possesses anti-inflammatory, antioxidant, and anti-cancer activities, its application in clinical settings is still hindered by several challenges. To date, no clinical studies have specifically evaluated the therapeutic effects of kaempferol in patients with CRC, and the current evidence is mainly derived from *in vitro* and *in vivo* experimental studies. One of the main obstacles limiting the clinical translation of kaempferol is its relatively low oral bioavailability, which is mainly attributed to poor aqueous solubility, limited intestinal absorption, and extensive first-pass metabolism in the intestine and liver (Qian et al., 2016; Zabela et al., 2016). Although kaempferol and its metabolites can be detected in systemic circulation and tissues after oral administration, experimental studies indicate that circulating concentrations remain relatively low, which may restrict its therapeutic efficacy in its native form (Barve et al., 2009; Zabela et al., 2016). To overcome this limitation, researchers are exploring advanced delivery technologies. Strategies like nanoencapsulation, liposomal carriers, and engineered exosomes are under development to improve kaempferol absorption, stability, and targeted delivery to tumor tissues (Ilk et al., 2017). Additionally, utilizing food industry byproducts as a sustainable source for kaempferol extraction offers a cost-effective alternative for pharmaceutical production (Parveen et al., 2023). In terms of safety, kaempferol is generally well tolerated. At standard doses, it does not harm normal cells and may even help protect against damage caused by chemotherapy (Akiyama et al., 2023). However, very high concentrations might cause oxidative stress, highlighting the need to define optimal dosing levels. Long-term studies are necessary to fully evaluate its safety and establish clinical guidelines (Sousa et al., 2021). The effects of kaempferol as an adjuvant in cancer therapy is also promising.When used with conventional drugs such as 5-FU or oxaliplatin, it may boost treatment efficacy and reduce toxicity (Saha et al., 2024). Its ability to modulate key molecular pathways and immune responses further supports its value in combination regimens. Future clinical trials are essential to determine the best methods for delivering kaempferol and to confirm its benefits in patients. With continued innovation and testing, kaempferol may evolve from a laboratory compound into a reliable clinical tool for cancer therapy, especially when integrated into personalized treatment plans.

Another critical consideration is that numerous anticancer properties of kaempferol documented *in vitro* are noted at micromolar concentrations, which may surpass the plasma levels attainable through typical dietary consumption or standard oral administration (Chen and Chen, 2013). Experimental investigations have frequently illustrated antiproliferative, pro-apoptotic, and anti-inflammatory effects at concentrations between approximately 10–100 μM within CRC cell lines (Chen and Chen, 2013; Imran et al., 2019). Nevertheless, pharmacokinetic studies reveal that kaempferol experiences rapid metabolic processes and extensive conjugation, leading to relatively diminished systemic bioavailability subsequent to oral intake (Calderón-Montaño et al, 2011; Dabeek and Marra, 2019). Therefore, although kaempferol exhibits promising biological activities, achieving therapeutically effective concentrations *in vivo* remains a major challenge. This limitation further highlights the importance of developing optimized delivery systems, such as nanoparticles, liposomes, and exosome-based formulations, to enhance its stability, absorption, and tissue-targeting capacity (Alam et al., 2020; Bangar et al., 2023).

## 9 Conclusion

CRC continues to pose a major global health burden, with rising incidence and persistent challenges such as drug resistance and metastatic progression. Despite advancements in conventional therapies, limitations in efficacy and safety have created a need for complementary treatment approaches. In this context, kaempferol, a naturally occurring flavonoid found in many edible and medicinal plants, has emerged as a promising candidate due to its ability to target multiple cancer-related pathways with minimal toxicity to normal cells. It exerts potent effects by modulating oxidative stress, suppressing chronic inflammation, inducing apoptosis, regulating the cell cycle, and reversing metabolic adaptations typical of cancer cells. Importantly, kaempferol has shown the capacity to re-sensitize resistant CRC cells to standard chemotherapeutic agents such as 5-FU and oxaliplatin. Despite promising preclinical evidence, the clinical application of kaempferol remains limited because of poor systemic bioavailability, rapid metabolism, and low water solubility. Emerging delivery strategies, including nanotechnology-based systems and combination therapies, may improve its therapeutic efficacy. Future studies should prioritize *in vivo* validation, pharmacokinetic characterization, and well-designed clinical trials to establish its efficacy, safety, and optimal dosing. Overall, kaempferol represents a promising multi-target natural compound with potential applications as a nutraceutical or adjuvant agent in CRC management.
